# Clinical and Epidemiological Aspects on Healthcare-Associated Infections with *Acinetobacter* spp. in a Neurosurgery Hospital in North-East Romania

**DOI:** 10.3390/medicina61060990

**Published:** 2025-05-27

**Authors:** Nicoleta Luchian, Iulia Olaru, Alina Pleșea-Condratovici, Mădălina Duceac (Covrig), Mirela Mătăsaru, Marius Gabriel Dabija, Eva Maria Elkan, Vlad Andrei Dabija, Lucian Eva, Letitia Doina Duceac

**Affiliations:** 1 Faculty of Medicine and Pharmacy, Doctoral School of Biomedical Sciences, “Dunarea de Jos” University of Galati, 47 Domnească Street, 800008 Galati, Romania; nicoletaluchian13@yahoo.com (N.L.); matasarumirela@gmail.com (M.M.); 2 Faculty of Medicine and Pharmacy, “Dunarea de Jos” University of Galati, 47 Domnească Street, 800008 Galati, Romania; iulia_dabija@yahoo.com (I.O.); cojocarumariaeva@yahoo.com (E.M.E.); elucian73@yahoo.com (L.E.); letimedr@yahoo.com (L.D.D.); 3 “Prof. Dr. N. Oblu” Clinic Emergency Hospital, 2, Ateneului Street, 700309 Iasi, Romania; marius.dabija@umfiasi.ro; 4 Department of Radiology and Medical Imaging, Faculty of Medicine, “Grigore T. Popa” University of Medicine and Pharmacy, 16, Universității Street, 700115 Iasi, Romania; vlad-andrei.dabija@email.umfiasi.ro

**Keywords:** healthcare-associated infections, *Acinetobacter*, neurosurgery, epidemiology, intensive care unit

## Abstract

*Background and Objectives*: Healthcare-associated infections (HAIs) are on the rise worldwide because the range of etiologic agents involved is very diverse and their antimicrobial resistance poses a threat to population health in the third millennium. *Materials and Methods*: We conducted a retrospective, longitudinal, descriptive clinical–epidemiological study in a hospital with a neurosurgical profile in northeastern Romania (“Prof. Dr. N. Oblu” Clinical Emergency Hospital in Iasi), during 2020–2024. The study is centered on the involvement of *Acinetobacter* spp. in the occurrence and evolution of HAIs. *Results*: The highest incidence of *Acinetobacter* spp. HAIs was recorded in the intensive care unit (ICU)—82.78% compared to neurosurgical wards (15.38%), with predominance in males (69.23%) and rural residence patients (55.67%). Most HAIs were represented by ventilator-associated pneumonia (42.13%) and lower respiratory tract infections (23.08%). Strains with high virulence and pathogenicity (CR-MDR, ESBL-MDR) were found, with the highest proportion of CR-MDR strains (88.27%). *Conclusions*: Our study provides useful data for surveillance of the antimicrobial resistance of pathogens involved in HAIs at the hospital level and for guiding antibiotic therapy in hospital-acquired infections.

## 1. Introduction

Healthcare-associated infections (HAIs) are among the priority public health problems due to the consequences they generate, as a result of specific morbidity and mortality, but also by creating the conditions for the emergence of multiple resistant microorganisms. HAIs are still largely underdiagnosed and underreported in Romania; it is necessary to know the real incidence of HAIs, as well as antibiotic resistance and antibiotic use, in order to calibrate interventions to limit the undesirable effects of HAIs. A healthcare-associated infection is defined as an infection that meets one of the European surveillance case definitions (as per European Commission Decision (EU) 2018/945) with onset of symptoms on day 1–3 of the current hospitalization or later (date of admission is considered as day 1) [1].

A 2022 WHO report (WHO, Global Report on Infection Prevention and Control) concludes that globally, for every 100 acute care inpatients, 22 patients worldwide will acquire at least one healthcare-associated infection during their stay. Up to 30% of patients in intensive care units may be affected by HAIs. Surveys on the prevalence of HAIs in the Eastern Mediterranean Region and the EU showed a prevalence of 11.2% and 6.5% in 2017 and 2018, respectively. The same report indicates that in EU countries in 2020, carbapenem resistance for *Klebsiella pneumoniae*, *Pseudomonas aeruginosa*, and *Acinetobacter* spp. was on average 10%, 17.8%, and 38%, respectively, and ECDC noted that the negative impact of the COVID-19 pandemic on antimicrobial resistance (AMR) is becoming increasingly visible in EU countries, especially for healthcare-associated pathogens; for example, in 2020, the carbapenem resistance of *Acinetobacter* spp. was equal to or above 50% in 55% of countries, especially in southern and eastern Europe [2].

A review that also observed an increase in the reporting of HAIs in Romania since the adoption of relevant legislation in 2016 found that the reported prevalence is still far from the European average of 7.1%, and has been underreported or underestimated over time, such that a 2018 European study found a 2.6% prevalence of HAIs at the national level [3,4].

The etiology of HAIs is extremely varied, encompassing Gram-positive and Gram-negative germs, with the pathogens being hospital-selected strains or introduced from the general population and most commonly antibiotic-resistant bacteria. Etiologic agents may be of endogenous or exogenous origin, by different modes and routes of transmission. HAIs are particularly prevalent and severe in high-risk groups. The emergence of multidrug-resistant strains due to the overuse of antibiotics and decontaminants poses challenges in the detection, surveillance, and control of HAIs [5]. Among the Gram-negative germs that thrive in the hospital environment, especially in intensive care units (ICUs), and generate HAIs with high pathogenicity is *Acinetobacter* spp.

Morphologic and physiologic characteristics evidence that *Acinetobacter* spp. present as short bacilli/cocobacils, usually 1.0–1.5 μm by 1.5–2.5 μm in size, measured during the rapid growth phase, usually present in pairs or chains of variable lengths [6,7,8]. The genus *Acinetobacter* comprises saprophytic, strictly aerobic, non-motile, encapsulated, saprophytic species [9,10], with a wide natural habitat including moist soil, wastewater, and water treatment plants.

The most common species of the genus found in the hospital microbial background are *A. baumanii*, *A. lwoffii*, *A. haemolyticus*, and *A. calcoaceticus*. The majority of *A. baumannii* infections involve organ systems containing high fluid levels such as the urinary and respiratory tract and the peritoneal cavity related to internal devices. Differentiating between infection and colonization with *A. baumannii* is difficult. The detection of *A. baumannii* in hospitalized patients is thought to be a sign of serious disease, with an associated mortality of approximately 30% [6].

As clinical entities of HAIs with *Acinetobacter* spp. we list nosocomial pneumonia frequently occurring in ICUs [11,12]; urinary tract infections, especially in urinary catheterized patients [13]; sepsis [14,15]; and nosocomial post-neurosurgical meningitis caused by multidrug-resistant strains, representing an increasingly important problem [7,16] as mortality can reach up to 70%, although the cause is often difficult to determine [17,18]. *Acinetobacter* MDR poses a threat in ICUs in mechanically ventilated patients [19,20].

The ability of the *A. baumannii* strain to develop multidrug resistance and to persist in harsh environmental conditions makes *Acinetobacter* spp. infections very dangerous, especially in patients who have recently undergone major surgery or have malignant disease or burns [21,22], or in immunocompromised patients such as the elderly and patients with diabetes mellitus, anemia [23], or HIV/AIDS [24].

Overuse of antibiotics, including some broad-spectrum or stock antibiotics, wrongly administered as first-line therapy, has resulted in *Acinetobacter* spp. strains acquiring multiple antimicrobial resistance by various mechanisms [25,26,27]. An important characteristic of *A. baumannii* is its tendency to cause epidemic outbreaks due to its resistance to antimicrobial agents and its ability to resist dehydration [17,28].

In terms of hospital equipment contamination with *Acinetobacter* spp., most reports refer to respiratory equipment used for mechanical ventilation, suctioning, and devices related to intravascular approach and access [29]. The hands of healthcare personnel may be colonized with strains of *A. baumannii*, thus facilitating spread to patients [30,31]. Epidemiologic studies demonstrated that rates of hand carriage among nurses and physicians ranged from 3% to 23% and were usually transient. [21,32,33].

Patients on mechanical ventilation, especially long-term patients, with longer hospital or ICU stays and with a higher degree of exposure to other infected or colonized patients in the nearby hospital environment, are at increased risk of acquiring multidrug-resistant strains [34].

In Romania, available data indicate a high and increasing level of bacterial resistance for some species more frequently implicated in HAIs, and the proportion of antibiotic-resistant isolates is still one of the highest in Europe [35,36]. The etiopathogenic mechanisms of HAIs reveal a diverse palette of causative agents—*Klebsiella* spp., *Acinetobacter* spp., *Pseudomonas* spp., *Proteus*, etc.—both in adult and pediatric hospitals, resulting in various entities of HAIs, with the involvement of the phenomenon of microbial antibiotic resistance [36,37].

Over time, *Acinetobacter* spp. has shown multidrug resistance to an extensive number of antibiotics: cephalosporins (ceftazidim or cefepim), carbapenems (imipenem or meropenem), ampicillin-sulbactam, fluoroquinolones (ciprofloxacin or levofloxacin), tetracyclines and glycyclines, and aminoglycosides (gentamicin, tobramycin or amikacin) [17,38,39,40,41]. Colistin is still the active antibiotic in the vast majority of these infections, and tobramycin is a possible solution for inhaled administration in patients with *A. baumannii* pneumonia [42].

Currently, efforts are being made to find innovative solutions based on nanoparticles that can fulfill the role of transporting and delivering antibiotics to target organs and tissues, which would contribute to faster cures and limit nosocomial phenomena [42,43,44].

### Aim and Objectives

This work aims to describe and analyze the incidence and distribution of healthcare-associated infections (HAIs) with etiology represented by *Acinetobacter* spp. strains in the wards of a hospital with a neurosurgical profile, over a four-year period (2020–2024), focusing on the identification of trends and potential preventive measures. The objectives are to determine the incidence of HAIs caused by strains of *Acinetobacter* spp.; socio-demographic analysis of cases of *Acinetobacter* spp., with particularization by patients’ backgrounds, gender, and age groups; analysis of the period of hospitalization from admission to the onset of HAIs; and evaluation of the antibiotic resistance profiles of *Acinetobacter* spp. strains, in particular *A. calcoaceticus* and *A. lwofii* isolated from infected patients.

## 2. Materials and Methods

### 2.1. Study Design and Hospital Context

The “Prof. Dr. Nicolae Oblu” Clinical Emergency Hospital of Iasi, Romania, was founded in 1972 by professor of neurosurgery Nicolae Oblu. The hospital is the largest and the only monospecialty hospital with a neurosurgery profile in northeastern Romania, with a wide reach to all localities of the region and the whole country. It is a university clinic hospital specializing in neurosurgery, neurology, ophthalmology, neuro-ophthalmology, intensive care, radiology, neuroimaging, functional examinations, and specialized laboratories. It is also a top university hospital, with a Center of Neuro Excellence at the national level. The hospital operates on six floors with 321 beds: Neurosurgery Clinic I—70 beds; Neurosurgery Clinic II—61 beds; Neurosurgery Clinic III—25 beds; Neurology Clinic I—50 beds; Neurology Clinic II—40 beds; Ophthalmology Clinic—45 beds; ICU—30 beds; and Day Hospital Stay—10 beds. Hospital statistics for the period 2020—2024 showed that the total number of hospitalized patients was 42,851, and the total number of HAIs was 1653 cases (3.85% of total inpatients) (Table 1).

This study was conducted at the “Prof. Dr. N. Oblu” Clinical Emergency Hospital, Iași, Romania, a neurosurgical hospital with clinical wards of Neurology (N1 and N2), Neurosurgery (NC1, NC2 and NC3), and ICU, during the period 1 January 2020–31 December 2024.

Our study was a retrospective descriptive study, including cases of HAIs with isolates positives for *Acinetobacter* spp.

### 2.2. Information Resources

In carrying out this study, information extracted from medical documents (observation sheets, medical registers, HAI charts, statistical reports, HAI register of HAI surveillance, and control service of that hospital) was used.

Inclusion criteria in the study: any age (since the hospital also admits pediatric patients from the region; in pediatric hospitals there is currently no pediatric neurosurgery department); diagnosis of HAI with *Acinetobacter* spp.; and signing of informed consent form by the adult patient or relatives for pediatric patients.

The diagnosis of HAIs with *Acinetobacter* spp. was made taking into account the microbiological result of the culture from the pathological product (tracheal aspirate; cerebrospinal fluid; secretion from the surgical wound/drain tube; blood culture; secretions from the superficial pressure ulcer; urine culture; skin secretion at the catheter implantation site/catheter tip culture). As a time interval, these were sampled as follows: at least 72 h after hospitalization if clinical signs appeared or for patients with ventilator-associated pneumonia (VAP) if the patient with pneumonia presented an invasive respiratory device, even temporarily, 48 h before the onset of pneumonia; for superficial surgical site infections, the infection was considered to have occurred within a maximum of 30 days after the surgical intervention.

Exclusion criteria: Patients who did not present a diagnosis of HAI with Acinetobacter; those who did not sign the informed consent form.

### 2.3. Antibiotic Susceptibility Testing

Antibiotic susceptibility testing was performed using the Kirby–Bauer diffusimetric method on Mueller–Hinton culture medium and the microdilution method for determining the MIC for Vancomycin and Colistin according to laboratory standards.

The following antibiotic microtablets were used for antibiotic susceptibility testing of *Acinetobacter calcoaceticus* strains by the diffusimetric method: Ampicillin/Sulbactam (AMS; 10/10 µg), Piperacillin/Tazobactam (TZP; 100/10 µg), Ceftazidime (CAZ; 30 µg), Ceftriaxone (CRO; 30 µg), Imipenem (IPM; 10 µg), Meropenem (MEM; 10 µg), Ciprofloxacin (CIP 5 µg), Levofloxacin (LEV 5 µg), Gentamicin (G 10 µg), Amikacin (AK 30 µg), Tobramycin (TOB 10 µg), and Trimethoprim/Sulfamethoxazole (TMP/SXT 1.25/23.75 µg). For some strains, the MIC for Colistin was determined. Confirmation of carbapenemase-resistant strains was achieved by determining resistance to IPM 10 µg and/or MEM 10 µg. Multidrug resistance was defined according to ECDC (lack of sensitivity to at least one antibiotic from 3 or more classes of antibiotics).

### 2.4. Statistical Analysis

The information material was processed according to various socio-demographic, etiological, clinical, and epidemiological parameters, and the results were interpreted using classical epidemiological, statistical–mathematical, and computerized methods. Patient data were anonymized and collected in Microsoft Office Excel and then processed.

### 2.5. Ethical Considerations

Formal approval for the study was obtained from the Hospital Ethics Committee, and the general provisions of the Declaration of Helsinki on Medical Research Involving Human Subjects were followed. Data collection started by searching the database of the health facility and selecting cases of patients who contracted HAIs during hospitalization.

The identification of cases of HAIs was performed by active or passive screening according to the case definitions according to clinical, epidemiological, and laboratory considerations, specified in Ministry of Health MS Order 1101/2016, with reference to Decision 2012/506/EU.

## 3. Results

The incidence of HAIs with *Acinetobacter* spp. in the period 2020–2024 had two peaks: 8.14% in 2021 and 7.92% in 2024.

### 3.1. Distribution by Wards

**Figure 1:**
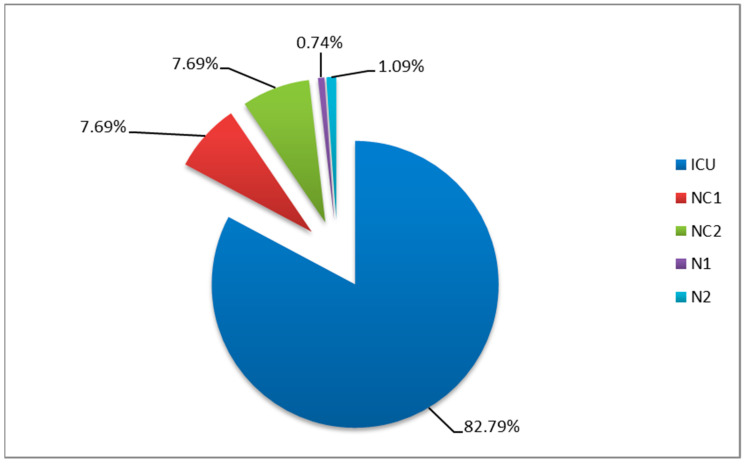
Distribution by wards of HAI isolates positive for *Acinetobacter* spp. (NC1, NC2—neurosurgical wards; N1, N2—neurological wards; ICU—intensive care unit).

The “Prof. Dr. N. Oblu” Clinical Emergency Hospital in Iasi, Romania, is a hospital with a neurosurgery profile with two neurosurgical clinics (NC1 and NC2), two neurology clinics (N1 and N2), and an ICU. Our 5-year study was conducted between 1 January 2020 and 31 December 2024. A total of 273 cases of HAIs with *Acinetobacter* spp. were identified. The biggest provider of HAI isolates positive for *Acinetobacter* spp. was ICU, with 226 cases (82.79%), followed by the NC1 and NC2 wards with 7.69% in total, and the N1 and N2 wards with 0.74% and 1.09%, respectively (Figure 1).

### 3.2. Distribution by Demographic Characteristics

**Figure 2:**
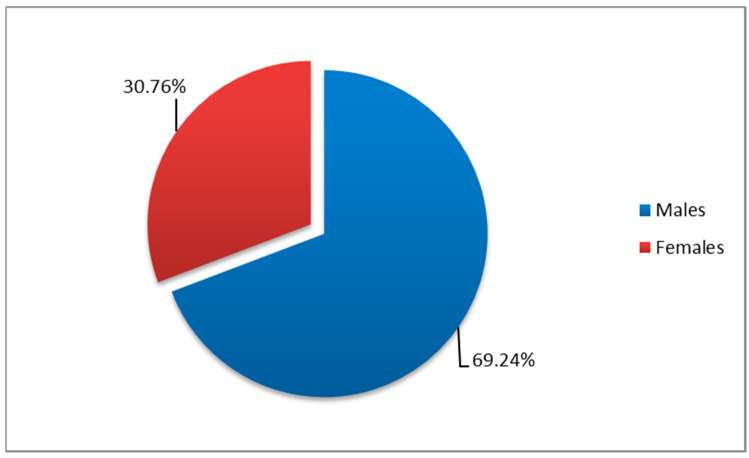
Gender distribution of HAI isolates positive for *Acinetobacter* spp.

Distribution by gender of HAI cases showed an M:F ratio = 2.25 (189 cases in males—69.24%; 84 cases in females—30.76%) (Figure 2).

**Figure 3:**
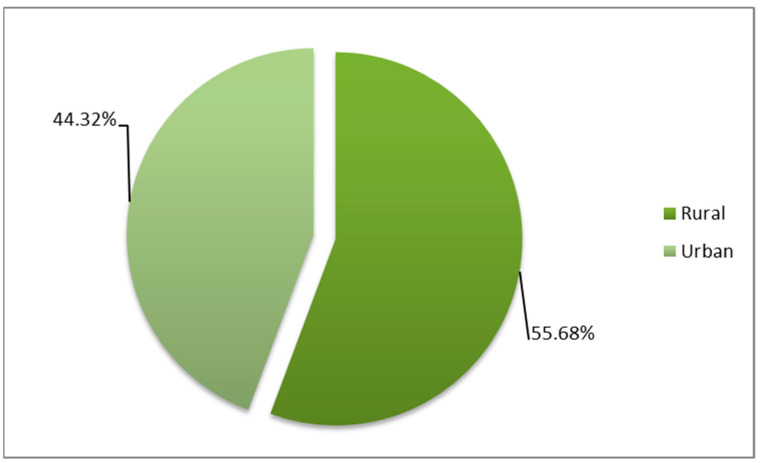
Distribution by origin areas of HAI cases positive for *Acinetobacter* spp.

According to the origin residence areas of the patients who developed *Acinetobacter* spp. HAIs, 121 patients, or 44.32%, came from urban areas and 152 patients, or 55.68%, from rural areas (Figure 3).

**Figure 4:**
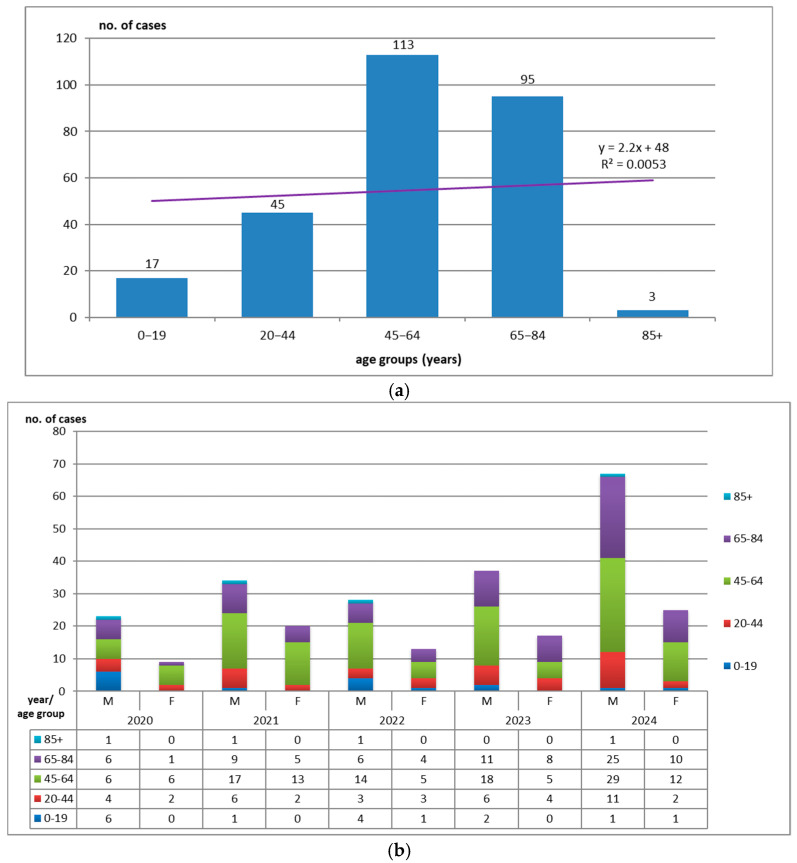
(**a**) Age histogram with linear trendline of the age of patients with HAIs positive for *Acinetobacter* spp. (R^2^ or R square—coefficient of determination; *x*—independent variable; *y*—dependent variable; R^2^ shows how much of the variation in the dependent variable *y* is explained by the independent variable *x*). (**b**) Distribution of cases by age groups, years, and genders.

The age groups of patients diagnosed with HAIs positive for *Acinetobacter* spp. are illustrated in Figure 4a,b. The highest HAI rate is observed in the age group 45–64 years (113 patients, 41.39%), followed closely by the 65–84 years age group (95 patients—34.79%). The young adult group (20–44 years) was affected, with 45 patients, 16.48%. The extreme (0–19 years) and over-85 years groups recorded the lowest prevalence: 6.22%, 17 patients, and 1.09%, 3 patients, respectively. It seems that patients’ immunosuppression and comorbidities, as well as the diversity of medical maneuvers and hygienic deficiencies of the hospital environment, play an important role in the development of HAIs.

### 3.3. Hospitalization Days

**Figure 5:**
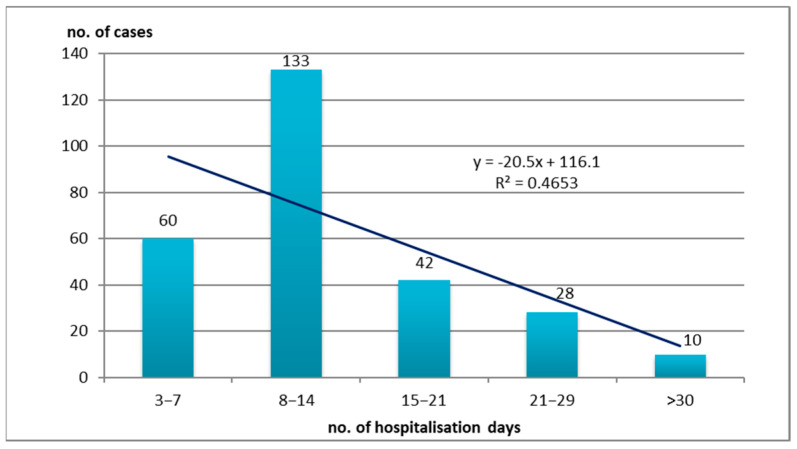
Hospitalization days of HAI cases with isolates positive for *Acinetobacter* spp. (R^2^ or R square—coefficient of determination; *x*—independent variable; *y*—dependent variable; R^2^ shows how much of the variation in the dependent variable *y* is explained by the independent variable *x*).

We were concerned with the estimation of the interval of hospitalization days of patients between contamination and the detection of HAIs. For this purpose, we grouped these intervals into smaller intervals of 3–7 days, 8–14 days, 15–21 days, 21–29 days, and more than 30 days (Figure 5).

Most HAIs (133 cases—48.72%) were contracted or detected within 8–14 days of hospitalization, another 60–21.98% within a shorter interval of 3–7 days, and another 42 cases (15.38%) were detected within 15–21 days of hospitalization. In a longer interval, 21–29 days, 28 HAIs were detected—10.25%. It can be observed that most HAIs were contracted by patients in the 8–14 days period (133 cases—40, 29%), although we would have expected that in the 21–29 days period and over 30 days the highest number of HAIs would have been recorded, as a longer period of hospitalization predisposes to a higher number of HAIs.

### 3.4. Clinical Entities

**Figure 6:**
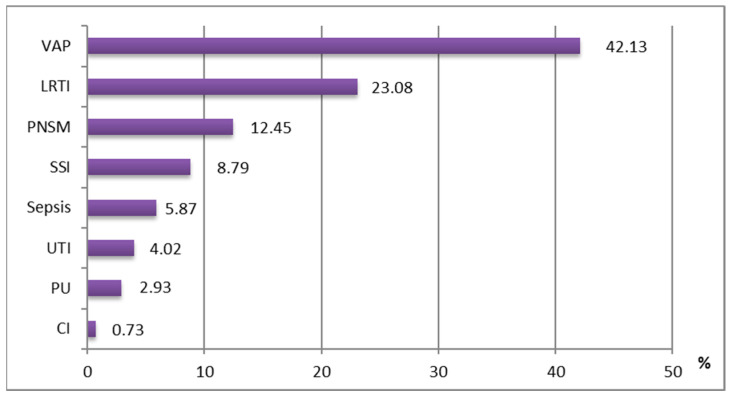
Proportion of clinical entities of HAI isolates positive for *Acinetobacter* spp. (error bars and standard deviation). (VAP—ventilator-associated pneumonia; LRTI—lower respiratory tract infection; SSI—surgical site infection; ITU—urinary tract infection; PU—pressure ulcer; CI—catheter infection).

In our study, we analyzed the clinical entities of HAIs with *Acinetobacter* spp. Most HAIs were represented by ventilator-associated pneumonias (VAP—115 cases, 42.13%) and lower respiratory tract infections (LRTI) (63 cases, 23.08%) with *A. calcoaceticus* or *A. lwoffii*, CR, MDR, ESBL, or combinations thereof, in the ICU. At the hospital level, there were 34 cases (12.45%) of post-neurosurgical meningitis (PNSM); 24 cases (8.79%) of superficial surgical site infections (SSIs); 16 cases (5.87%) of sepsis; 11 cases (4%) of urinary tract infections (UTIs); 8 cases (2.93%) of skin infections such as superficial pressure ulcers; and 2 cases (0.73%) of peripheral catheter infections (CIs) (Figure 6).

### 3.5. Antimicrobial Resistance

**Figure 7:**
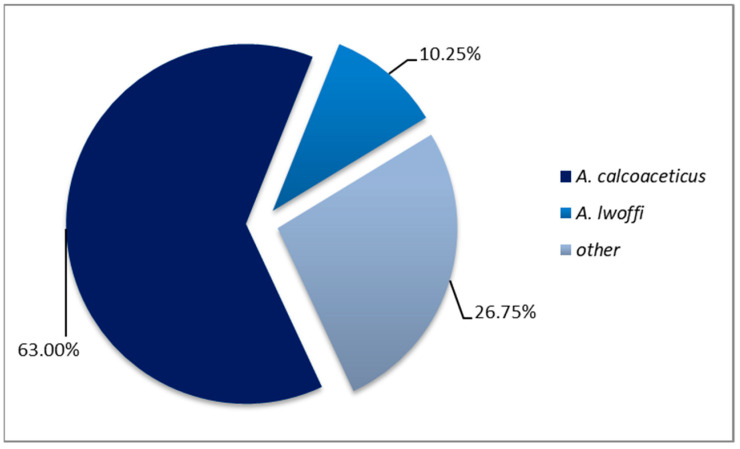
Proportion of HAI isolates positive for *A. calcoaceticus* and *A. lwoffi*.

The criterion of pathogenicity, in terms of antimicrobial resistance, was investigated by analyzing the results of microbial cultures, recorded and processed in the microbiology laboratory of the hospital. Strains isolated in the biological samples taken from the patients showed a predominance of *A. calcoaceticus* strains (172 isolates, 63%) and *A. lwoffii* strains (28 isolates, 10.25%) (Figure 7).

**Figure 8:**
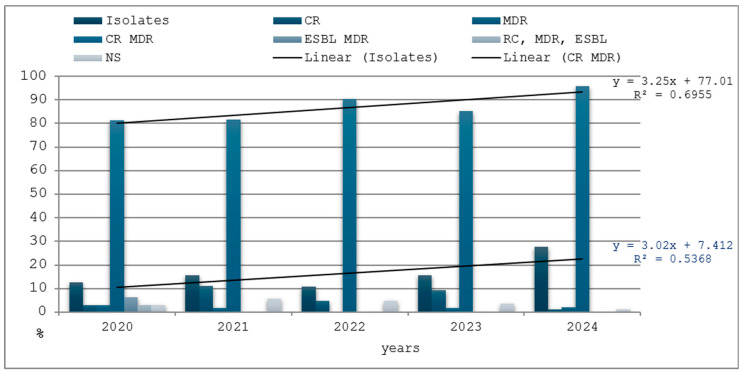
Antimicrobial resistance types of *Acinetobacter* spp. strains. (CR—carbapenem-resistant; MDR—multidrug-resistant (resistant to more than 3 classes of antibiotics); ESBL (extended-spectrum beta-lactamase); NS—non-specific.) (R2 or R square—coefficient of determination; *x*—independent variable; *y*—dependent variable; R2 shows how much of the variation in the dependent variable y is explained by the independent variable x).

Table 2 and Figure 8 illustrate the resistance of *Acinetobacter* spp. isolates to antimicrobials, such as CR (carbapenem-resistant), MDR (multidrug-resistant (resistant to more than 3 classes of antibiotics)), and ESBL (extended-spectrum beta-lactamase enzyme secreting), and combinations of them, all with a broad spectrum of pathogenicity.

It can be observed that the CR-MDR strains (241 cases, 88.27%) account for the highest proportion, but the most virulent strains represented 0.36% of the total (CR MDR ESBL).

## 4. Discussion

Healthcare-associated infections, often caused by antimicrobial-resistant pathogens, can be considered the most common adverse outcome as a result of healthcare delivery.

Globally, regionally, and nationally, healthcare-associated infections represent both a serious population health problem and a significant economic burden, particularly for areas with limited health resources. It is estimated that at any given time, 7% of patients will acquire at least one HAI and about 10% of these cases will result in death. No healthcare system in the most developed countries can claim to be free of cases of HAIs [19,45,46].

In the European Union, the prevalence of patients with HAIs is 6% (with a range of 2.3–10.8% between countries). The European Centre for Disease Prevention and Control (ECDC) estimates that, on average, HAIs affect 1 in 20 hospitalized patients, which represents 4.1 million people annually in the EU, and that each year such infections generate 25 million additional days of hospitalization, cause 37 thousand deaths, cause an additional 110 thousand deaths, and cost an estimated 13–24 billion euros annually [46]. The risk of acquiring HAIs in healthcare institutions in developing countries is 2 to 20 times more frequent than in developed countries [34].

Regarding the distribution by wards and by demographic characteristics, our research revealed that the highest incidence rates of HAIs with *Acinetobacter* spp. were recorded in the ICU (82.78%), with a predominance of male patients (69.23%) and rural residence patients (55.67%), most frequently in the 45–64 age group (41.39%).

In our hospital, the separate wards of NC1, NC2, N1, and N2 have independent medical staff. Each ward has its own physicians, nurses, and auxiliary health personnel. The circuits also are separate, with these wards operating on different floors (from the second to the fifth floor; on the first floor is the ICU ward, and on the sixth floor is the Ophthalmology ward that has its operating room on the same floor). What is common between the NC1 and NC2 wards is only the operating rooms, which are located on the first floor, but in a different hospital wing from the ICU, which operate in relation to appointments and emergencies. Therefore, we considered that the separate distribution of cases by wards could highlight some risk factors for HAIs, such as those related to human error and the experience of medical and auxiliary staff in applying HAI prevention measures (asepsis, antisepsis, and disinfection).

A cross-sectional study [38], conducted in the west-central part of Romania, on HAI cases, in a general public hospital, highlighted that, regarding the distribution of cases by residence environment, more than half came from urban areas rather than from rural areas, in contrast to our study group, where more cases came from rural areas (55.67%). Although the differences in frequency are not significant, the area of residence could be listed among the demographic variables when analyzing a sample of HAI cases. For a more detailed analysis, other variables such as educational level, emergency or not, diagnosis, and comorbidities would be necessary. We consider the idea worthy of study in future research.

The analysis of hospitalization days has shown that the detection of these HAIs was mostly within 8–14 days (40.29%).

Concerning the distribution by clinical entities, the most common in-hospital infections were ventilator-associated pneumonias (VAPs), 42.67%, and lower respiratory tract infections (LRTIs), 23.08%.

We also studied the antimicrobial resistance, which highlighted that the etiologic spectrum was dominated by the subtype *A. calcoaceticus* (63%), mostly CR-MDR (88.27%), as indubitable evidence of pathogenicity.

In this study, we sought to compare our study with others conducted at the national level. For instance, the Current Prevalence Study of Healthcare-Associated Infections and Antimicrobial Consumption, conducted as part of the large ECDC-PPS study in hospitals in Romania, a study undertaken by INSP in 2023 [42], highlighted a total of 725 reported HAIs, of which the etiological agent was identified for 626 (86.3%), resulting in a total of 720 identified microorganisms. The main microorganisms involved in the etiology of HAIs identified in that study were as follows: the first place was occupied by *Clostridioides difficile* (26%), followed by *Klebsiella* spp. (17%), *Pseudomonas aeruginosa* (9%), and *Acinetobacter* spp. (9%). In the etiology of respiratory infections, the predominant microorganisms were *Acinetobacter* spp. (22.3%) and SARS-CoV-2 (22.3%), followed by *Klebsiella* spp. (18.7%) and *P. aeruginosa* (16.9%) [47].

From the compared data, it is found that the respiratory HAIs in our study also have *Acinetobacter* spp. as an etiology (23.07%).

A retrospective observational study [47] on the incidence of *A. baumannii* infections in the ICU of the “Victor Babeș” Hospital for Infectious Diseases and Pneumophthisiology Timișoara, Romania, during the period June 2011–June 2015 identified a high incidence of *A. baumannii* infections, with an average hospitalization period of 6 days.

As shown by the statistical analysis, prolonged hospitalization was not necessarily a risk factor for contracting HAIs, but we can consider other factors such as older age, some neurosurgical diagnoses, comorbidities, and associated immunosuppression. We mention that these will be the objective of future research conducted in our hospital.

Bronchial aspiration was the most frequent pathological cause in this study (90% of cases). Antimicrobial resistance was also determined: the lowest resistance was recorded for ampicillin + sulbactam (81.1%), and the highest resistance rate was recorded for ceftazidime and imipenem (94.6% each). Comparing resistance to third-generation cephalosporins, the difference was not statistically significant (94.6% for ceftazidime versus 86.5% for cefoperazone, *p* = 0.117). In the study, a significantly high resistance of the germ to carbapenems was observed, with good sensitivity to aminoglycosides and Colistin. Only one strain of *A. baumannii* was resistant to all classes of antibiotics tested.

It should also be noted that during the period 2020–2024, corresponding to our study, the COVID-19 pandemic also evolved, which increased the risk of nosocomial infections through various mechanisms (immunosuppression and comorbidities of patients infected with the SARS-CoV-2 virus, antibiotic abuse due to fear of severe superinfections, prolonged hospitalizations, etc.), making the work of medical personnel more difficult [48,49]. A comparative study states that *A. baumannii* infections in patients with COVID-19 have a substantially increased mortality, reaching a percentage of 85.7%. At the same time, the presence of multidrug-resistant strains of this pathogen worsens the prognosis, being associated with an even higher mortality among infected patients. This analysis emphasizes the severity of co-infection and its unfavorable impact on clinical evolution [49].

The universal spread of *Acinetobacter* spp. is illustrated by the fact that several epidemiological studies have reported the occurrence of MDR *A. baumannii* infections in different regions of the world: in Europe, the Eastern Mediterranean, Africa [50], North America, Central Asia (Turkey, Iraq, Afghanistan), East Asia (China, Taiwan, Hong Kong, Japan and Korea) [51], Argentina, and Brazil [52], often associated with nosocomial infections [50,51,52].

Although aseptic technology is utilized at a modern level, patients who have undergone neurosurgical intervention have an increased risk of non-infectious or infectious meningitis, and bacterial meningitis can have life-threatening consequences. According to recently published studies and case reports, the incidence of post-neurosurgical meningitis has varied between 0.3 and 10% following various neurosurgical interventions [53,54].

In our study, we reported an incidence of 12.45% over a 5-year period. Post-neurosurgical bacterial meningitis is not a common infection; therefore, we consider that multicenter, national, and international prospective studies are necessary to improve the reporting of this condition as an HAI, and to adapt hospital management policies in this regard in order to ensure the safety of the neurosurgical patient.

Current practices are the general ones mentioned in the national protocols for the prevention of HAIs, being related to the observance of circuits, asepsis, antisepsis, and disinfection measures. These are the same for the entire hospital and do not differ by department; what differs is the medical staff, who may be subject to human errors, given that the cases can be serious, and the fact that the hospital also provides neurological and neurosurgical emergencies throughout the northeastern region of the country. All of these could certainly have contributed to influencing the HAI incidence values.

Study limitations: In our opinion, the present study is unique in the neurosurgery hospital profile regarding the etiology of *Acinetobacter* spp. nosocomial infections that may occur during the hospitalization of patients. Currently, the national epidemiological surveillance system of HAIs, based on active and passive reporting, does not provide consistent and standardized data for the identification of risk factors, epidemiological monitoring of causative pathogens of HAIs, or planning of infection control measures. We consider it essential to improve the epidemiological surveillance and control system of HAIs at the level of all hospitals to make it more efficient in predicting trends and evaluating the effectiveness of the measures taken. In our opinion, this study represents a beginning in the research of HAIs with *Acinetobacter* in neurosurgical hospitals, and this fact limited us in comparing the results obtained with other similar studies.

Specific study limitations could be related to the fact that this study was conducted in a single hospital, which may limit the generalizability to settings with different patient populations and infection control or neurosurgical protocols. Since we used a cross-sectional design, there was no control group, and the study aim itself was limited to the epidemiology field. The classical statistical analyses used can be added to the list of limitations, as well as the short period of time including the years of the COVID-19 pandemic.

## 5. Conclusions

Our study highlighted cases of HAIs with *Acinetobacter* spp., a severe infection in patients with neurosurgical interventions. Prevention of this infection is important and can be achieved as successfully as possible by correctly applying antisepsis and disinfection measures and respecting hospital protocols and circuits. Also, by limiting the occurrence of such cases, we can also limit the occurrence of increasingly severe antibiotic resistance in *Acinetobacter* spp. strains. We believe that our study brings pioneering novelty and thus opens the way for more extensive research in the future.

## Figures and Tables

**Table 1:** Statistical data on the number of patients and the number of cases of HAIs including isolates positive for *Acinetobacter* spp., during 2020–2024.

Years	No. of Patients	HAIs Cases		HAIs with *Acinetobacter* spp.		
		No.	%	No.	%	Incidence * (‰)
2020	5786	255	4.41	32	12.55	5.53
2021	6632	347	5.23	54	15.56	8.14
2022	8467	375	4.43	41	10.93	4.84
2023	10,343	342	3.31	54	15.78	5.22
2024	11,623	334	2.37	92	27.54	7.92
Total	42,851	1653	3.85	273	16.51	6.37

* *p* = 0.083.

**Table 2:** Distribution by year of AMR types of isolates from HAIs positive for *Acinetobacter* spp.

Years	Isolates		CR		MDR		CR MDR		ESBL MDR		CR, MDR, ESBL		NS	
	No.	%	No.	%	No.	%	No.	%	No.	%	Nr.	%	No.	%
2020	32	12.55	1	3.12	1	3.12	26	81.25	2	6.25	1	3.12	1	3.12
2021	54	15.56	6	11.11	1	1.85	44	81.48	0	0.00	0	0.00	3	5.55
2022	41	10.93	2	4.87	0	0.00	37	90.24	0	0.00	0	0.00	2	4.87
2023	54	15.78	5	9.25	1	1.85	46	85.18	0	0.00	0	0.00	2	3.70
2024	92	27.54	1	1.08	2	2.17	88	95.65	0	0.00	0	0.00	1	1.08
Total	273	16.51	15	5.49	5	1.83	241	88.27	2	0.73	1	0.36	9	3.29

Legend: CR—carbapenem-resistant; MDR—multidrug-resistant (resistant to more than 3 classes of antibiotics); ESBL (extended-spectrum beta-lactamase); NS—non-specific.

## Data Availability

All data are available from the first author and the corresponding author.

## Abbreviations

The following abbreviations are used in this manuscript:

HAI	Healthcare-associated infection
CR	Carbapenem resistance
MDR	Multidrug resistance
ESBL	Extended-spectrum beta-lactamase
ICU	Intensive care unit
